# Association Between Physical Literacy and Quality of Life Among Japanese Adults: The Mediating Role of Moderate to Vigorous Physical Activity

**DOI:** 10.3390/healthcare14162468

**Published:** 2026-08-10

**Authors:** Donghai Xu, Misaki Matsunaga, Masahiro Matsui, Kenta Toyama, Yibo Gao, Koya Suzuki

**Affiliations:** 1 Graduate School of Health and Sports Science, Juntendo University, Chiba 270-1695, Japan; xudonghai1998@outlook.com (D.X.);; 2 Institute of Health and Sports Science & Medicine, Juntendo University, Chiba 270-1695, Japan; 3 Juntendo Administration for Sports, Health and Medical Sciences, Tokyo 138-8421, Japan; m.matsunaga.of@juntendo.ac.jp; 4 Faculty of Health and Sports Science, Juntendo University, Chiba 270-1695, Japan; masahiromatsui0708@gmail.com (M.M.); wingatektoyama@gmail.com (K.T.); 5 Department of Child Psychiatry, Yokohama City University Hospital, Kanagawa 236-0004, Japan

**Keywords:** physical literacy, quality of life, moderate-to-vigorous physical activity, assessment tool, mediation analysis

## Abstract

**Background/Objectives:** Physical literacy (PL) is increasingly being recognized as an important factor associated with physical activity participation and health outcomes. However, evidence remains limited regarding the association between PL and quality of life (QOL) in adults, particularly the statistical indirect association through moderate-to-vigorous physical activity (MVPA). Therefore, this cross-sectional study aimed to examine the associations among PL, MVPA, and QOL in Japanese adults and to assess the statistical indirect association between PL and QOL through MVPA. **Methods:** Japanese adults aged 18–64 years were recruited from a commercial online survey panel using age- and sex-quota sampling in 2023. After excluding incomplete or inconsistent responses and participants with missing or incomplete MVPA data, 861 participants were ultimately included. PL, QOL, and MVPA were assessed using the Physical Literacy for Life tool, World Health Organization QOL-26, and International Physical Activity Questionnaire, respectively. Associations among PL, MVPA, and QOL were examined using Pearson’s correlations, multiple linear regression, and mediation analysis with PROCESS macro (Model 4). **Results:** PL was positively correlated with MVPA (r = 0.257, *p* < 0.01) and QOL (r = 0.416, *p* < 0.01); MVPA was positively correlated with QOL (r = 0.248, *p* < 0.01). PL remained significantly associated with QOL after including MVPA and covariates (β = 0.374, *p* < 0.01). The statistical indirect association between PL and QOL through MVPA was significant (b = 0.0011, BootSE = 0.0003, 95% bootstrap confidence intervals: 0.0005–0.0018), indicating a modest indirect association through MVPA. **Conclusions:** PL was positively associated with QOL among Japanese adults, and MVPA partially explained this association in a statistical mediation model.

## 1. Introduction

The World Health Organization (WHO) defines health as a state of complete physical, mental, and social well-being. In this context, Quality of life (QOL) has gained increasing attention as an important indicator of the multidimensional state of health. The WHO defines QOL as an individual’s perception of their position in life in the context of the culture and value systems in which they live and in relation to their goals, expectations, standards, and concerns [1]. This concept indicates that health is not merely the absence of disease or the preservation of functional capacity but rather a comprehensive state encompassing physical, psychological, and social dimensions. Lower QOL is associated with psychological problems such as depression and anxiety [2], as well as increased risks of hospitalization and all-cause mortality [3]. Therefore, QOL is an important outcome in health promotion research and practice.

In recent years, Physical literacy (PL) has attracted growing scholarly attention as an important health-related concept [4,5]. PL refers to individuals’ motivation, confidence, physical competence, knowledge, and understanding to value and take responsibility for engaging in physical activity (PA) throughout life [6]. Empirical evidence shows that higher PL is positively associated with better QOL among university students and that this relationship was partially mediated by psychological factors such as motivation and satisfaction in physical education classes [7]. Additionally, PL is associated with perceived health, subjective well-being, life satisfaction, and psychological distress [8]. Taken together, existing theoretical and empirical evidence support the notion that PL is an important determinant of health and well-being [9,10]. Thus, enhancing PL may contribute to better QOL through multiple physical, psychological, cognitive, and social pathways.

Among the potential pathways linking PL to QOL, moderate-to-vigorous physical activity (MVPA) is widely recognized as a key health-promoting behavior [11]. The WHO recommends that adults aged 18–64 years accumulate at least 150–300 min of moderate-intensity physical activity, 75–150 min of vigorous-intensity physical activity, or an equivalent combination of both per week to obtain substantial physical and mental health benefits [12]. In addition, a large body of evidence has shown that higher levels of MVPA are associated not only with lower risks of chronic diseases [13], but also with better mental health, subjective well-being, and QOL [14]. Therefore, MVPA may represent an important behavioral pathway through which PL is related to QOL.

From a theoretical perspective, PL can be regarded as a distal, multidimensional factor related to PA behavior because it includes motivation, confidence, physical competence, knowledge, and understanding for lifelong engagement in PA [15]. In this framework, MVPA may represent a proximal behavioral pathway linking PL with broader health-related outcomes such as QOL. Previous conceptual and empirical studies have suggested that PL is associated with health outcomes through PA behavior and that higher PL is related to greater MVPA or overall PA participation [16]. Therefore, examining MVPA as a statistical mediator is theoretically reasonable. However, because this study used cross-sectional data, the proposed model should be interpreted as a theory-driven statistical model rather than evidence of a causal sequence.

Although previous studies have examined the associations between PL and QOL, the available evidence remains limited in scope. For example, Wang et al. [7] investigated the relationship between PL and QOL among university students and reported that motivation played a mediating role in this association. The study provided important evidence for a psychological pathway linking PL to QOL; however, owing to the study’s focus on a student population and on motivation, which is closely embedded within the affective dimension of PL, the association of PL with QOL through actual health-related behavior in the broader adult population remains undetermined. Moreover, most previous studies have focused on pairwise associations between PL and PA, or between PL and selected indicators of mental health and well-being [9]. Therefore, little is known about how PL, MVPA, and QOL are interrelated within a single conceptual model in the general adult population. This knowledge gap is significant because PL is a multidimensional construct that includes not only physical competence but also motivational, cognitive, affective, and social components. Therefore, the association between PL and QOL may be explained only partly by engagement in MVPA, while other psychological, cognitive, and social dimensions of PL may also be relevant to QOL.

To address this gap, the present study examined the associations among PL, MVPA, and QOL in Japanese adults aged 18–64 years and assessed the statistical indirect association between PL and QOL through MVPA. Rather than simply replicating previously reported associations in a different population, this study aimed to clarify whether MVPA accounts for a significant part of the PL–QOL association and determine the extent to which this association remains unexplained by MVPA. Based on previous theoretical and empirical evidence, we hypothesized that higher PL would be positively associated with better QOL and that this association would be partly explained by MVPA.

## 2. Materials and Methods

### 2.1. Participants

The present study was conducted as a secondary analysis of data collected during the same online survey wave conducted in November 2023 that was used in our previous validation study of the Japanese version of the Physical Literacy for Life (PL4L) questionnaire [17]. Therefore, participants in the present study overlapped with those included in Matsunaga et al. (2025). However, the present analysis addressed a different research question and used a different analytic sample after applying the PA data-processing criteria.

Participants were recruited from a commercial online survey panel operated by a major Japanese online survey company (MyVoice Communications, Inc., Tokyo, Japan) [18] which had approximately 3.35 million registered panel members. Between 1 and 6 November 2023, the survey company recruited 1132 Japanese adults aged 18–64 years using age- and sex-based quotas. Before delivering the dataset to the research team, the survey company first excluded 124 respondents because of incomplete responses or logical inconsistencies identified through its quality-control procedures. As part of the company’s sampling and data-delivery procedure, 68 participants were then randomly removed from overrepresented age-by-sex strata to achieve approximately equal numbers across strata. This quota adjustment was performed without reference to the study variables or outcomes, including PL, MVPA, and QOL. The resulting dataset containing 940 respondents was then delivered to the research team. After receiving the dataset, the research team excluded an additional 79 respondents because their IPAQ-SF data were missing or insufficient for calculating MVPA according to the prespecified data-processing criteria. The final analytic sample therefore comprised 861 participants.

### 2.2. Measures

#### 2.2.1. Physical Literacy

PL was assessed using the Japanese Physical Literacy for Life self-assessment tool (PL4L). The PL4L comprises 16 items across four domains: six items in the physical domain, four in the emotional domain, three in the cognitive domain, and three in the social domain. Each item is scored on a three-point scale from 0 to 2. The PL4L comprises four domains: physical (6 items; possible raw score range, 0–12), emotional (4 items; 0–8), cognitive (3 items; 0–6), and social (3 items; 0–6). For each domain, the raw score is divided by the maximum possible score for that domain and multiplied by 100, yielding a standardized domain score ranging from 0 to 100. The total PL score is calculated as the arithmetic mean of the four standardized domain scores and therefore also ranges from 0 to 100. The total PL score was used as the primary explanatory variable because the PL4L is designed to assess PL as a multidimensional yet integrated construct, and the total score reflects the overall level of PL across the physical, emotional, cognitive, and social domains. Higher scores indicate higher levels of PL. The psychometric properties of the Japanese version of the PL4L were previously evaluated using data from a survey wave that overlapped with the dataset used in the present study. In that study, the scale demonstrated satisfactory model fit (RMSEA = 0.053, GFI = 0.955, AGFI = 0.937, CFI = 0.970, and AIC = 435.124), with standardized factor loadings ranging from 0.56 to 0.85 [17]. In the present analytic sample (n = 861), the 16-item PL4L demonstrated excellent internal consistency (Cronbach’s α = 0.921; McDonald’s ω = 0.921). The corresponding reliability coefficients were α = 0.887 and ω = 0.886 for the physical domain; α = 0.760 and ω = 0.758 for the emotional domain; α = 0.829 and ω = 0.835 for the cognitive domain; and α = 0.873 and ω = 0.873 for the social domain.

#### 2.2.2. Quality of Life

QOL was assessed using the Japanese version of the 26-item WHO Quality of Life questionnaire (WHOQOL-BREF/QOL26) [19]. The original WHOQOL-BREF was developed by the WHO as an internationally applicable measure of QOL and comprises 26 items, including 24 items covering four domains—physical health, psychological health, social relationships, and environment—and 2 global items assessing overall QOL and general health [20]. The Japanese version was developed through linguistic translation and cultural adaptation to ensure that the wording and content were appropriate for Japanese populations. This questionnaire has demonstrated good internal consistency among Japanese adults, with Cronbach’s α coefficients for the subscales of 0.66–0.84 [19].

Each item was rated on a five-point scale ranging from 1 to 5. Items 3, 4, and 26 were reverse coded so that higher scores consistently indicated better QOL. In accordance with the scoring convention of the Japanese QOL26 manual [19], the overall QOL score used as the primary outcome was calculated as the mean of all 26 items, including the 24 domain-specific items and the two global items assessing overall QOL and general health. Domain scores were calculated as the mean of the corresponding items: physical health (items 3, 4, 10, 15, 16, 17, and 18), psychological health (items 5, 6, 7, 11, 19, and 26), social relationships (items 20, 21, and 22), and environment (items 8, 9, 12, 13, 14, 23, 24, and 25). Both the overall and domain scores ranged from 1 to 5, with higher scores representing better QOL.

In the present sample, the 26-item summary score demonstrated excellent internal consistency (Cronbach’s α = 0.937; McDonald’s ω = 0.937). The corresponding reliability coefficients were α = 0.777 and ω = 0.766 for the physical health domain; α = 0.845 and ω = 0.848 for the psychological health domain; α = 0.744 and ω = 0.749 for the social relationships domain; and α = 0.828 and ω = 0.824 for the environmental domain.

#### 2.2.3. MVPA

MVPA was assessed using the Japanese short version of the International Physical Activity Questionnaire (IPAQ-SF). In the present study, MVPA was calculated as the sum of the weekly time spent performing moderate-intensity and vigorous-intensity physical activity, excluding walking, and was expressed as hours per week (h/week). MVPA was used as a continuous variable in all analyses.

Data cleaning and processing were performed in accordance with the official Guidelines for Data Processing and Analysis of the IPAQ [21]. For the retained analytic sample, reported daily durations of walking, moderate-intensity activity, and vigorous-intensity activity were truncated at 180 min per activity category per day, in accordance with the standard IPAQ protocol. Walking was considered only during data cleaning and was not included in the calculation of MVPA. Additionally, we confirmed that no participant in the final analytic sample had a combined total PA duration exceeding 960 min per day. The reliability and validity of the IPAQ have been established across 12 countries, including Japan, with intraclass correlation coefficients of 0.75–0.80 [22].

Weekly MVPA was calculated as the sum of time spent in moderate- and vigorous-intensity physical activity and expressed in hours per week. Vigorous-intensity minutes were not additionally weighted because the primary exposure represented actual accumulated time spent in MVPA rather than energy expenditure or guideline-equivalent activity. Therefore, this variable should not be interpreted as IPAQ MET min/week or as a direct measure of WHO physical activity guideline attainment.

#### 2.2.4. Covariates

Age, sex, education, and annual income were included as covariates. Age was treated as a continuous variable, and sex was coded as 1 for men and 2 for women. Education was coded as 0 for without a college education and 1 for college graduates. Annual income was treated as an ordinal variable and coded from 1 to 10, with higher values indicating higher annual income.

### 2.3. Data Analysis

All the statistical analyses were performed using SPSS version 29.0.2.0 (IBM Corp., Armonk, NY, USA). The distributions of continuous variables were assessed using the Kolmogorov–Smirnov test and visual inspection. Continuous variables were presented as means ± standard deviations, whereas categorical variables were presented as frequencies and percentages. Statistical significance was set at *p* < 0.05.

Between-group comparisons were performed using independent-samples *t*-tests. Associations between variables were examined using Pearson’s correlation coefficient and multiple linear regression analysis adjusted for covariates including age, sex, education, and annual income. Pearson correlation coefficients were reported as descriptive bivariate associations, whereas 95% confidence intervals (CIs) were reported for the primary regression and statistical mediation model estimates.

In the regression analyses, multicollinearity was assessed using variance inflation factors (VIFs), with VIF values of < 10 indicating the absence of problematic multicollinearity [23]. As PL, MVPA, and QOL were assessed using self-reported questionnaires within the same survey, Harman’s single-factor test was conducted as an exploratory post hoc diagnostic of potential common method variance [24]. However, this test was not regarded as sufficient to establish the presence or absence of common method bias. Mediation analysis was performed using the PROCESS macro for SPSS version 4.0, Model 4, with parameter estimates based on 5000 bias-corrected bootstrap samples. In this analysis, the total PL score was defined as the exposure, MVPA as the mediator, and total QOL score as the outcome. The indirect effect was considered statistically significant when the 95% confidence interval (CI) did not include zero.

To assess the robustness of the fully adjusted regression estimates to potential heteroskedasticity, the final regression model was additionally estimated using HC3 heteroskedasticity-consistent standard errors. Furthermore, to describe subgroup-specific associations between PL and QOL, stratified regression analyses were conducted by sex and age group. Sex-stratified models were adjusted for age, education, and annual income, whereas age-stratified models were adjusted for sex, education, and annual income. No formal interaction tests were conducted.

## 3. Results

The Kolmogorov–Smirnov test indicated that the key variables did not significantly deviate from a normal distribution (all *p* > 0.05). In addition, the absolute skewness values were <2 and the absolute kurtosis values were <7 for all key variables, further supporting the assumption of approximate normality. Therefore, the normality assumption required for the subsequent parametric analyses was considered to be satisfied. Multicollinearity diagnostics showed that the VIF values for all independent variables ranged from 1.061 to 2.515, all of which were below 10, indicating no evidence of problematic multicollinearity in the regression models [25]. In the exploratory Harman single factor analysis, the first factor in the unrotated solution accounted for 30.16% of the total variance. This result indicated that no single factor accounted for most of the observed variance. However, Harman’s single-factor test is a limited diagnostic tool and cannot rule out the possibility of common method variance.

### 3.1. Descriptive Statistics

After excluding participants with missing or incomplete IPAQ-SF data, the final analytic sample comprised 861 participants, including 428 men (49.7%) and 433 women (50.3%). Overall, 477 participants (55.4%) were college graduates. The mean age of the participants was 41.76 ± 13.20 years, with no significant difference between men and women (42.04 ± 13.25 vs. 41.48 ± 13.16 years, *p* = 0.545). Men reported significantly higher annual income than did women (4.07 ± 2.64 vs. 2.07 ± 1.71, *p* < 0.001) and significantly higher MVPA (4.24 ± 4.00 vs. 3.54 ± 3.63 h/week, *p* = 0.007). Men also scored significantly higher than women across all PL domains and on the total PL score (all *p* < 0.05). Regarding QOL, women scored significantly higher than men in the social relationships domain (3.07 ± 0.69 vs. 2.90 ± 0.83, *p* < 0.001), whereas no significant sex differences were observed in the physical health, psychological health, environmental, or overall QOL scores (all *p* > 0.05) (Table 1).

Education status (college graduation) was treated as a binary variable (non-college graduate or college graduate) and analyzed separately using a chi-square test. Overall, 477 participants (55.4%) were college graduates. The proportion of college graduates was significantly higher among men than among women (61.4% vs. 49.4%; χ^2^(1) = 12.599, *p* < 0.001, φ = 0.121).

### 3.2. Correlation Analysis

Correlation analysis revealed a significant positive association between total PL and total QOL (r = 0.416, *p* < 0.01). All PL subdomains were also positively correlated with total QOL, including the physical (r = 0.281, *p* < 0.01), emotional (r = 0.438, *p* < 0.01), cognitive (r = 0.358, *p* < 0.01), and social (r = 0.306, *p* < 0.01) domains. Additionally, total PL score was positively correlated with MVPA (r = 0.257, *p* < 0.01). Similarly, all PL subdomains were positively correlated with MVPA, including the physical (r = 0.192, *p* < 0.01), emotional (r = 0.269, *p* < 0.01), cognitive (r = 0.197, *p* < 0.01), and social (r = 0.205, *p* < 0.01) domains.

MVPA was positively correlated with total QOL (r = 0.248, *p* < 0.01) and all QOL subdomains, including the physical (r = 0.227, *p* < 0.01), psychological (r = 0.207, *p* < 0.01), social (r = 0.150, *p* < 0.01), and environmental (r = 0.245, *p* < 0.01) domains. Education was positively correlated with MVPA (r = 0.085, *p* < 0.05), total PL (r = 0.135, *p* < 0.01), and total QOL (r = 0.130, *p* < 0.01). Annual income was also positively correlated with MVPA (r = 0.139, *p* < 0.01), total PL (r = 0.288, *p* < 0.01), and total QOL (r = 0.173, *p* < 0.01). Furthermore, sex was negatively correlated with both MVPA (r = −0.092, *p* < 0.01) and total PL (r = −0.137, *p* < 0.01) but was not significantly correlated with total QOL. Age was not significantly correlated with MVPA, total PL, or total QOL. However, age was negatively correlated with the physical domain of PL (r = −0.148, *p* < 0.01) and positively correlated with the emotional domain of PL (r = 0.068, *p* < 0.05), the psychological domain of QOL (r = 0.116, *p* < 0.01), and annual income (r = 0.192, *p* < 0.01) (Table 2).

### 3.3. Analysis of Mediation Effects

Hierarchical multiple linear regression analyses were conducted to examine the associations of PL and MVPA with QOL, adjusting for sex, age, education, and annual income.

Model 1 was statistically significant (F = 8.255, *p* < 0.001) and explained 4.2% of the variance in QOL. Annual income was positively associated with QOL (b = 0.042, 95% CI: 0.021–0.062; β = 0.166; *p* < 0.001), whereas education was negatively associated with QOL (b = −0.126, 95% CI: −0.216 to −0.035; β = −0.101; *p* = 0.007). Sex and age were not significantly associated with QOL. Although education was positively correlated with QOL in the bivariate analysis (r = 0.130, *p* < 0.01), its coefficient became negative after adjustment for age, sex, and annual income in Model 1 (b = −0.126, β = −0.101, *p* = 0.007). This sign reversal may be compatible with a suppression effect or shared variance between education and annual income, which were moderately positively correlated (r = 0.303, *p* < 0.01). However, the present analysis was not designed to determine the underlying mechanism of this coefficient change. The negative coefficient for education was attenuated after PL and MVPA were entered into subsequent models.

After PL was entered in Model 2, the explained variance increased to 19.7%. PL was positively associated with QOL (b = 0.014, 95% CI: 0.011–0.016; β = 0.407; *p* < 0.001). In addition, sex and annual income were also positively associated with QOL, whereas age was not significantly associated with QOL. The association with education was borderline but did not meet the prespecified significance threshold (*p* = 0.050).

After MVPA was additionally entered in Model 3, the model explained 21.2% of the variance in QOL. Both PL (b = 0.012, 95% CI: 0.010–0.015; β = 0.374; *p* < 0.001) and MVPA (b = 0.021, 95% CI: 0.011–0.032; β = 0.131; *p* < 0.001) were positively associated with QOL. Sex and annual income remained significantly associated with QOL, whereas age and education were not significant (Table 3). As a robustness analysis, the fully adjusted regression model was re-estimated using HC3 heteroskedasticity-consistent standard errors. The results were materially unchanged. PL remained positively associated with QOL (b = 0.012, HC3 SE = 0.001, 95% CI: 0.010–0.015, *p* < 0.001), as did MVPA (b = 0.021, HC3 SE = 0.006, 95% CI: 0.010–0.032, *p* < 0.001), supporting the robustness of the primary regression findings.

**Figure 1:**
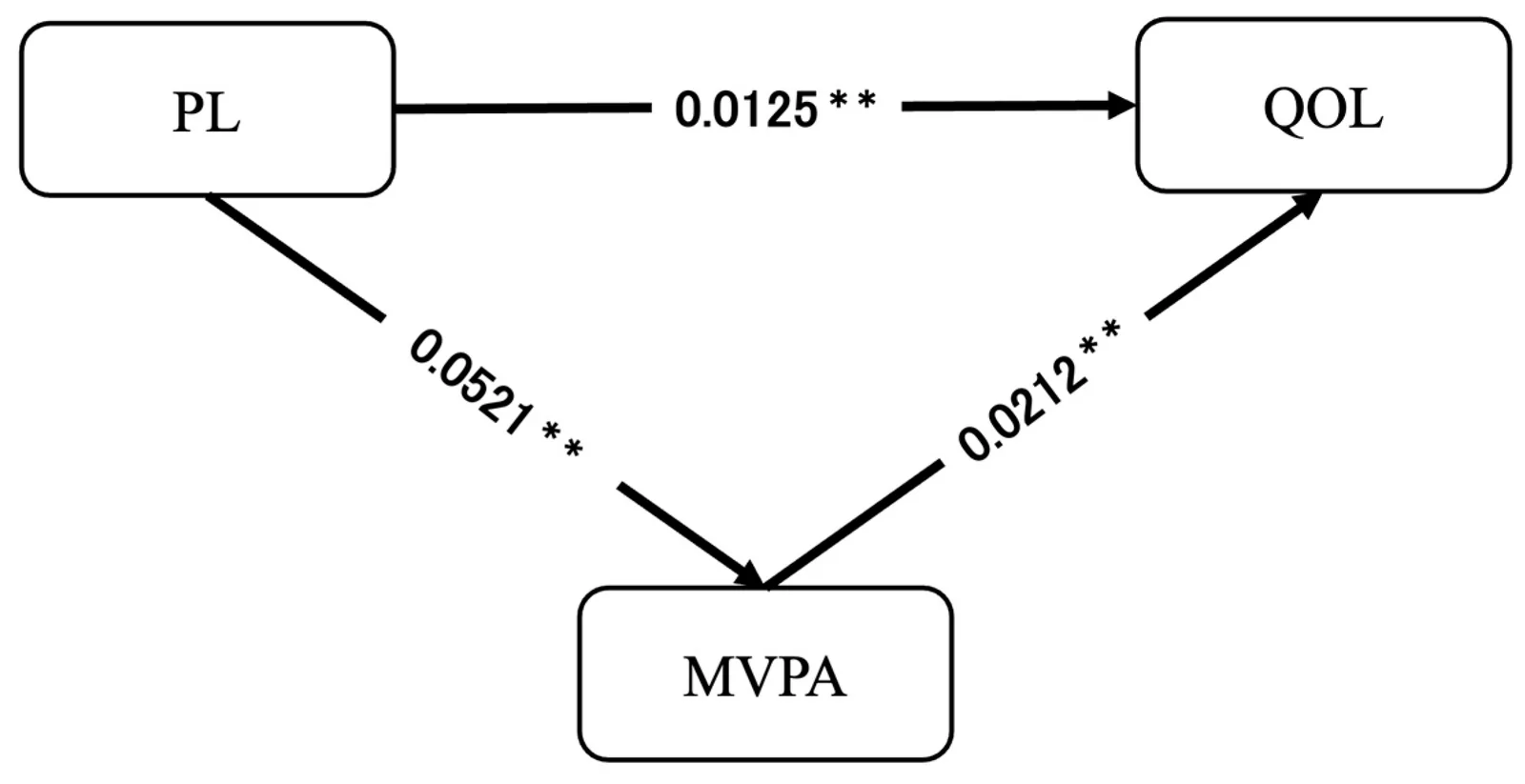
Statistical mediation model of MVPA in the association between PL and QOL. Note: Values on the paths are unstandardized coefficients. The model was adjusted for age, sex, income, and education. The total association between PL and QOL was b = 0.0136, 95% CI: 0.0114–0.0158. The indirect association through MVPA was b = 0.0011, BootSE = 0.0003, 95% bootstrap CI: 0.0005–0.0018, accounting for 8.09% of the total association. PL, physical literacy; MVPA, moderate-to-vigorous physical activity; QOL, quality of life. ** *p* < 0.001.

Furthermore, the statistical indirect association through MVPA was examined using the PROCESS macro for SPSS, adjusting for age, sex, education, and annual income. The total association between PL and QOL was statistically significant (b = 0.0136, SE = 0.0011, 95% CI: 0.0114–0.0158, *p* < 0.001). After MVPA was included in the model, the direct association between PL and QOL remained statistically significant (b = 0.0125, SE = 0.0011, 95% CI: 0.0102–0.0147, *p* < 0.001). In addition, the indirect association between PL and QOL through MVPA was also statistically significant (b = 0.0011, BootSE = 0.0003, 95% bootstrap CI: 0.0005–0.0018), as the bootstrap CI did not include zero. This indirect association accounted for 8.09% of the total association, indicating that MVPA explained a relatively small proportion of the association between PL and QOL (Table 4, Figure 1).

Domain-specific sensitivity analyses yielded a consistent pattern across the four QOL domains. Statistically significant indirect associations through MVPA were observed for physical health (b = 0.0011, 95% bootstrap CI: 0.0004–0.0019), psychological health (b = 0.0009, 95% bootstrap CI: 0.0002–0.0018), social relationships (b = 0.0010, 95% bootstrap CI: 0.0002–0.0019), and environment (b = 0.0013, 95% bootstrap CI: 0.0006–0.0021). Although the magnitude varied modestly across domains, all bootstrap CIs excluded zero (Table S1).

Stratified analyses revealed positive associations between PL and QOL within both men (β = 0.422, 95% CI for b: 0.011–0.017, *p* < 0.001) and women (β = 0.367, 95% CI for b: 0.010–0.016, *p* < 0.001). Positive within-group associations were also observed among adults aged 18–29 years (β = 0.523), 30–39 years (β = 0.364), 40–49 years (β = 0.298), and 50–64 years (β = 0.331) (all *p* < 0.001). These subgroup-specific estimates are descriptive and should not be interpreted as evidence of differences in association strength between groups because no formal interaction tests were conducted (Table 5).

## 4. Discussion

This study examined the associations between PL, MVPA, and QOL in Japanese adults using a mediation model. The results showed that higher PL was significantly associated with better QOL, and MVPA accounted for a small but statistically significant indirect association between PL and QOL. These findings suggest that PL may be linked to QOL both directly and indirectly through engagement in MVPA.

### 4.1. Association Between PL and QOL

PL was positively associated with QOL among Japanese adults. In the mediation analysis, the direct association between PL and QOL accounted for 91.91% of the total association, suggesting that the relationship between PL and QOL may not be explained solely by MVPA. This finding is consistent with the conceptual model, which positions PL as an upstream determinant of health and suggests that PL may be associated with multidimensional physical, psychological, and social health outcomes through its relationship with PA [4].

Recent studies have reported associations between PL or its related components and QOL. For example, physical competence, which is an important component of PL, has been associated with QOL. In addition, interventions designed to enhance movement-related affordances in children and adolescents improved PL and QOL scores, suggesting a potential relationship between improved PL and better QOL [26]. Furthermore, a previous study reported a significant positive association between PL and QOL, which was partially mediated by motivation, suggesting that the motivational dimension of PL may contribute to individuals’ perceived value and meaning of physical activity and health [7]. Consistent with these findings, the present study demonstrated a positive association between PL and QOL in the general adult population.

From a theoretical perspective, the association between PL and QOL may reflect the multidimensional nature of PL. PL includes not only physical competence but also affective, cognitive, and social dimensions. The affective dimension, including motivation and confidence, may be related to more positive self-perceptions of physical capability and health [27]. Individuals with higher motivation and confidence may be more likely to engage in adaptive coping behaviors when facing stress or health-related challenges, which could contribute to better mental health and subjective well-being [28]. The cognitive dimension of PL, including knowledge and understanding of physical activity and health, may help individuals recognize the importance of lifestyle behaviors, such as PA and sedentary behavior for long-term health and QOL [29]. In addition, the social dimension of PL, comprising communication, cooperation, and social engagement in movement-related contexts, may contribute to social connectedness and healthier lifestyles [30]. These multidimensional resources may help explain why PL was associated with QOL beyond its indirect association through MVPA. The relatively high correlations between the total PL score and its subdomains were expected because the total score was calculated from these domains. The moderate-to-high correlations among the PL subdomains reflect the conceptualization of PL as an integrated construct with interrelated physical, affective, cognitive, and social components. Therefore, the total PL score was used as the primary explanatory variable to represent overall PL and avoid interpretive difficulties arising from entering multiple related PL dimensions into the same model.

Notably, men scored higher than women across all PL domains and in the total PL score and a sex difference was also observed in the social domain of QOL. Although PL was positively associated with QOL in both sex groups and across all age groups, these stratified results should be interpreted cautiously because formal interaction tests were not conducted. Therefore, the present study cannot determine whether sex or age modified the association between PL and QOL. Future studies with larger and more diverse samples should further examine whether sex, age, socioeconomic status, or health conditions modify the relationship between PL and QOL.

### 4.2. Association Between PL, MVPA, and the Statistical Indirect Association Through MVPA

PL was significantly associated with MVPA, and MVPA was significantly associated with QOL. The mediation analysis further showed a statistically significant indirect association between PL and QOL through MVPA, accounting for 8.09% of the total association. These findings are consistent with the proposed theoretical model in which individuals with higher PL are more likely to engage in MVPA, which is associated with better QOL. However, the relatively small proportion of the association mediated by MVPA suggests that other psychological, cognitive, and social pathways may also be important.

The observed association between PL and MVPA is consistent with previous findings showing that adults with higher PL tend to report higher PA levels [16]. The four dimensions embedded in PL may explain the observed positive association between PL and MVPA. First, a higher PL level reflects a stronger foundation of basic movement skills [31]. Second, higher motor competence can effectively enhance an individual’s motivation to exercise, thereby facilitating greater participation in MVPA and serving as an important determinant of health behavior [32]. Additionally, individuals with higher PL levels tended to be more aware of the benefits of exercise, which is closely associated with higher levels of MVPA participation. More importantly, these multiple dimensions operate in an integrated and mutually reinforcing manner, forming a positive cycle through which PL functions as a powerful intrinsic driver of MVPA [4]. Thus, the present findings support the holistic conceptual framework of PL in fostering MVPA.

In addition, the results of the present study showed that MVPA was significantly associated with QOL. MVPA exerts broad and beneficial effects on physical health and perceived health status [33]. However, from psychological and social perspectives, regular participation in MVPA may help alleviate depressive symptoms and improve subjective well-being and life satisfaction. This finding has been confirmed in a systematic review by Marquez et al., which involved participants across different age groups [34]. The study revealed that, particularly among adults and older adults, an appropriate level of MVPA can significantly enhance overall QOL and psychological well-being. Mechanistically, MVPA improves sleep quality and daytime vitality through neuroendocrine pathways [35] while enhancing self-efficacy, thereby contributing to greater emotional stability and subjective well-being [36]. Taken together, these findings suggest that PL is associated with higher MVPA through multiple dimensions and that higher MVPA is associated with better individual health and well-being, which is reflected in higher QOL.

MVPA accounted for only 8.09% of the total association between PL and QOL. Although this indirect association was statistically significant, its magnitude was relatively small [37]. Therefore, MVPA should be interpreted as one limited behavioral pathway rather than the dominant explanation for the PL–QOL association. The larger remaining association may reflect other dimensions inherent in PL, such as motivation, confidence, perceived competence, health-related knowledge, and social engagement [4,38]. In addition, because QOL is a broad multidimensional outcome influenced by social and contextual factors [39,40], the proportion explained by a single behavioral variable such as MVPA is likely to be limited.

The domain-specific analyses further revealed small but statistically significant indirect associations through MVPA across all four QOL domains. Although their magnitudes varied modestly, the overall pattern was consistent across physical, psychological, social, and environmental QOL, suggesting that the observed statistical indirect association was not confined to a single QOL domain.

### 4.3. Limitations

This study has several limitations. First, because of its cross-sectional design, causal relationships among PL, MVPA, and QOL could not be inferred. Although the mediation model was based on theoretical and empirical evidence, the cross-sectional design prevented confirmation of the temporal ordering of PL, MVPA, and QOL. Therefore, the indirect association through MVPA should be interpreted as a statistical pattern rather than evidence of a causal pathway. Longitudinal and intervention studies are needed to examine whether improvements in PL lead to increased MVPA and subsequent improvements in QOL. Second, although the regression and mediation models were adjusted for age, sex, education, and annual income, residual confounding by other sociodemographic and health-related factors, such as employment status, marital status, chronic diseases, sleep, and other lifestyle factors, cannot be ruled out. Therefore, the observed associations should be interpreted cautiously. Third, MVPA was assessed using a self-report measure rather than device-based assessment, which may have introduced measurement error. Future studies should consider using accelerometers or other objective measures of PA. Fourth, PL, MVPA, and QOL were assessed using self-reported questionnaires administered within the same survey, which may have introduced common method variance and potentially inflated some of the observed associations. Although Harman’s single-factor analysis indicated that no single factor accounted for most of the variance, this test is a limited diagnostic and cannot rule out common method bias. In addition, no theoretically unrelated marker variable was pre-specified in the original survey design; therefore, an appropriate CFA marker-variable analysis could not be conducted retrospectively. Future studies should be aimed at incorporating a pre-specified marker variable, multiple data sources, temporal separation of measurements, or objective assessments where feasible. Finally, the survey company completed its quality-control exclusions and age-by-sex quota adjustment before delivering the dataset to the research team. Because individual-level records for respondents excluded by the company were not available, we could not compare included and excluded respondents or construct sampling weights to account for differential selection into the delivered sample. Consequently, although age- and sex-based quotas were applied, the final sample should not be regarded as a probability sample representative of the Japanese adult population, and the generalizability of the findings should be interpreted with caution.

## 5. Conclusions

This study demonstrated that, among Japanese adults, higher levels of PL were significantly associated with better QOL; additionally, MVPA accounted for a small but statistically significant indirect association between PL and QOL. Furthermore, the positive association between PL and QOL was observed across sex and age subgroups. These findings suggest that PL may be a relevant factor to consider in adult health promotion. However, given the cross-sectional design, longitudinal and intervention studies are needed to determine whether strategies targeting PL can increase MVPA or improve QOL.

## Figures and Tables

**Table 1:** Descriptive statistics and sex comparisons (n = 861).

Variables	All	Men	Women	t	*p*	Cohen’s d	95% CI	Skewness	Kurtosis
N (%)	861(100.0)	428 (49.7)	433 (50.3)						
Age (Years)	41.76 ± 13.20	42.04 ± 13.25	41.48 ± 13.16	0.606	0.545	0.041	−0.092, 0.175	−0.028	−1.177
Annual income	3.08 ± 2.45	4.07 ± 2.64	2.07 ± 1.71	12.395	<0.001	0.896	0.747, 1.044	1.190	0.644
MVPA (h/week)	3.89 ± 3.84	4.24 ± 4.00	3.54 ± 3.63	2.705	0.007	0.184	0.050, 0.318	1.180	0.818
PL (Score)									
Physical (Score)	55.82 ± 16.68	58.17 ± 18.85	53.50 ± 13.85	4.149	<0.001	0.283	0.148, 0.417	0.911	2.298
Emotional (Score)	55.77 ± 23.19	59.14 ± 24.58	52.45 ± 21.24	4.272	<0.001	0.291	0.157, 0.425	−0.147	0.122
Cognitive (Score)	51.49 ± 24.89	53.66 ± 26.27	49.34 ± 23.27	2.551	0.011	0.174	0.040, 0.308	0.115	0.116
Social (Score)	51.14 ± 22.76	53.42 ± 23.81	48.88 ± 21.47	2.940	0.003	0.200	0.066, 0.334	−0.001	1.094
PL Total (Score)	53.55 ± 18.47	56.10 ± 20.09	51.04 ± 16.35	4.051	<0.001	0.276	0.142, 0.410	0.352	1.030
QOL (Score)									
Physical (Score)	3.30 ± 0.65	3.35 ± 0.66	3.26 ± 0.63	1.861	0.063	0.127	−0.007, 0.261	−0.339	0.524
Psychological (Score)	3.02 ± 0.76	3.06 ± 0.78	2.99 ± 0.73	1.519	0.129	0.104	−0.030, 0.237	−0.119	−0.061
Social (Score)	2.98 ± 0.76	2.90 ± 0.83	3.07 ± 0.69	−3.345	<0.001	−0.228	−0.362, −0.094	−0.346	0.487
Environment (Score)	3.20 ± 0.63	3.21 ± 0.67	3.18 ± 0.58	0.612	0.541	0.042	−0.092, 0.175	−0.096	0.659
QOL Total (Score)	3.14 ± 0.60	3.16 ± 0.63	3.13 ± 0.56	0.725	0.469	0.049	−0.084, 0.183	−0.209	0.501

PL: Physical literacy; MVPA: Moderate-to-vigorous physical activity; QOL: Quality of life. Note: Annual income was treated as an ordinal variable and coded from 1 to 10 as follows: 1 = <2 million JPY; 2 = 2 to <3 million JPY; 3 = 3 to <4 million JPY; 4 = 4 to <5 million JPY; 5 = 5 to <6 million JPY; 6 = 6 to <7 million JPY; 7 = 7 to <8 million JPY; 8 = 8 to <9 million JPY; 9 = 9 to <10 million JPY; and 10 = ≥10 million JPY. An independent-samples *t*-test was used to compare men and women.

**Table 2:** Correlations between variables (n = 861).

	Age	Sex	Education	Annual Income	MVPA	PL-Phy	PL-Emo	PL-Cog	PL-Soc	PL	QOL-Phy	QOL-Psy	QOL-Soc	QOL-Env	QOL
Age	1.000														
Sex	−0.021	1.000													
Education	−0.049	−0.121 **	1.000												
Annual income	0.192 **	−0.409 **	0.303 **	1.000											
MVPA	−0.007	−0.092 **	0.085*	0.139**	1.000										
PL-Phy	−0.148 **	−0.140 **	0.053	0.157 **	0.192 **	1.000									
PL-Emo	0.068 *	−0.144 **	0.130 **	0.218 **	0.269 **	0.566 **	1.000								
PL-Cog	0.006	−0.087 *	0.116 **	0.207 **	0.197 **	0.543 **	0.679 **	1.000							
PL-Soc	0.012	−0.100 **	0.137 **	0.176 **	0.205 **	0.498 **	0.622 **	0.702 **	1.000						
PL	−0.006	−0.137 **	0.135 **	0.288 **	0.257 **	0.740 **	0.862 **	0.889 **	0.852 **	1.000					
QOL-Phy	0.039	−0.063	0.082 *	0.152 **	0.227 **	0.307 **	0.407 **	0.324 **	0.262 **	0.387 **	1.000				
QOL-Psy	0.116 **	−0.052	0.135 **	0.193 **	0.207 **	0.212 **	0.412 **	0.331 **	0.295 **	0.379 **	0.774 **	1.000			
QOL-Soc	−0.029	0.113 **	0.092 **	0.036	0.150 **	0.190 **	0.284 **	0.263 **	0.232 **	0.292 **	0.552 **	0.632 **	1.000		
QOL-Env	−0.016	−0.021	0.132 **	0.168 **	0.245**	0.228 **	0.366 **	0.305 **	0.269 **	0.352 **	0.679 **	0.715 **	0.608 **	1.000	
QOL	0.031	−0.025	0.130 **	0.173 **	0.248 **	0.281 **	0.438 **	0.358 **	0.306 **	0.416 **	0.888 **	0.912 **	0.745 **	0.884 **	1.000

PL: Physical literacy; PL-Phy: Physical domain of PL; PL-Emo: Emotional domain of PL; PL-Cog: Cognitive domain of PL; PL-Soc: Social domain of PL; MVPA: Moderate-to-vigorous physical activity; QOL: Quality of life; QOL-Phy: Physical domain of QOL; QOL-Psy: Psychological domain of QOL; QOL-Soc: Social domain of QOL; QOL-Env: Environment domain of QOL; * *p* < 0.05, ** *p* < 0.01.

**Table 3:** Hierarchical multiple linear regression models examining the associations of PL and MVPA with QOL (n = 861).

Variables	Model 1				Model 2				Model 3			
	b	SE	β	95% CI	b	SE	β	95% CI	b	SE	β	95% CI
Sex	0.064	0.048	0.052	−0.030, 0.158	0.099	0.044	0.081 *	0.013, 0.185	0.106	0.044	0.086 *	0.020, 0.191
Age	−0.001	0.002	−0.015	−0.004, 0.003	0	0.002	−0.003	−0.003, 0.003	0	0.002	0	−0.003, 0.003
Education	−0.126	0.046	−0.101 **	−0.216, −0.035	−0.083	0.042	−0.067	−0.167, 0.000	−0.077	0.042	−0.062	−0.160, 0.006
Income	0.042	0.01	0.166 **	0.021, 0.062	0.024	0.01	0.094 *	0.005, 0.042	0.022	0.01	0.086 *	0.003, 0.040
PL					0.014	0.001	0.407 **	0.011, 0.016	0.012	0.001	0.374 **	0.010, 0.015
MVPA, h/week									0.021	0.005	0.131 **	0.011, 0.032
F	8.255 **				37.198 **				34.111 **			
R^2^	0.042				0.197				0.212			
Adjusted R^2^	0.037				0.191				0.206			

b: unstandardized coefficient; SE: standard error; β: standardized regression coefficient; PL: physical literacy; MVPA: moderate-to-vigorous physical activity; QOL: quality of life; * *p* < 0.05, ** *p* < 0.01.

**Table 4:** Completely unstandardized direct and indirect associations of PL with QOL through MVPA (n = 861).

Mechanism	Effect	SE/BootSE	95% CI		Contribution Rate (%)
			LLCI	ULCI	
Total effect	0.0136	0.0011	0.0114	0.0158	100
Direct effect	0.0125	0.0011	0.0102	0.0147	91.91
Indirect effect: PL → MVPA → QOL	0.0011	0.0003	0.0005	0.0018	8.09

SE: standard error; PL: physical literacy; QOL: quality of life; MVPA: moderate-to-vigorous physical activity.

**Table 5:** Stratified multiple linear regression analysis of the association between PL and QOL.

Groups	n	b	SE	β	95% CI	R^2^
Men	428	0.014	0.002	0.422 **	0.011–0.017	0.218
Women	433	0.013	0.002	0.367 **	0.010–0.016	0.183
18–29 years	218	0.017	0.002	0.523 **	0.013–0.022	0.294
30–39 years	182	0.012	0.002	0.364 **	0.007–0.017	0.161
40–49 years	188	0.010	0.002	0.298 **	0.005–0.014	0.184
50–64 years	273	0.012	0.002	0.331 **	0.008–0.016	0.159

b: unstandardized coefficient; SE: standard error; β: standardized regression coefficient; PL: physical literacy; QOL: quality of life. Note: Models were adjusted for age, income, and education in sex-stratified analyses, and for sex, income, and education in age-stratified analyses. ** *p* < 0.001.

## Data Availability

All the data are available from the corresponding author upon reason-able request due to privacy restrictions.

## Supplementary Materials

The following supporting information can be downloaded at: https://www.mdpi.com/article/10.3390/healthcare14162468/s1, Table S1: Domain-specific statistical mediation analyses of the association between PL and QOL through MVPA.

## Abbreviations

The following abbreviations are used in this manuscript:

PL	Physical literacy
QOL	Quality of life
MVPA	Moderate-to-vigorous physical activity
PA	Physical activity
PL4L	Physical Literacy for Life self-assessment tool
WHO	World Health Organization
