# Supplementary material for: Association Between Physical Literacy and Quality of Life Among Japanese Adults: The Mediating Role of Moderate to Vigorous Physical Activity
Source: Healthcare (Basel). 2026 Aug 10;14(16):2468. doi: 10.3390/healthcare14162468 (PMC13512086; doi:10.3390/healthcare14162468)
Supplement: Supplementary file 1 [file healthcare-14-02468-s001.zip › healthcare-4381648-supplementary.pdf]

Table S1. Domain-specific statistical mediation analyses of the association between PL and QOL through MVPA

QOL domain	Total association, b (95% CI)	Direct association, b (95% CI)	MVPA → QOL, b (95% CI)	Indirect association, b	BootSE	Bootstrap 95% CI
Physical health	0.0137 (0.0113–0.0161)	0.0126 (0.0101–0.0151)	0.0211 (0.0093–0.0328)	0.0011	0.0004	0.0004–0.0019
Psychological health	0.0154 (0.0126–0.0182)	0.0145 (0.0116–0.0173)	0.0178 (0.0042–0.0315)	0.0009	0.0004	0.0002–0.0018
Social relationships	0.0128 (0.0099–0.0158)	0.0118 (0.0088–0.0148)	0.0196 (0.0053–0.0339)	0.0010	0.0004	0.0002–0.0019
Environment	0.0118 (0.0094–0.0142)	0.0105 (0.0081–0.0130)	0.0242 (0.0128–0.0356)	0.0013	0.0004	0.0006–0.0021

Note: Values are unstandardized coefficients. All models were adjusted for age, sex, education, and annual income. Indirect associations were estimated using 5,000 percentile bootstrap samples. PL, physical literacy; MVPA, moderate-to-vigorous physical activity; QOL, quality of life.
